# Quercetin Alleviated Inflammasome-Mediated Pyroptosis and Modulated the mTOR/P70S6/P6/eIF4E/4EBP1 Pathway in Ischemic Stroke

**DOI:** 10.3390/ph16081182

**Published:** 2023-08-21

**Authors:** Abdullah Alattar, Reem Alshaman, Yusuf S. Althobaiti, Ghareb M. Soliman, Howaida S. Ali, Waleed Salman Khubrni, Phil Ok Koh, Najeeb Ur Rehman, Fawad Ali Shah

**Affiliations:** 1 Department of Pharmacology and Toxicology, Faculty of Pharmacy, University of Tabuk, Tabuk 47713, Saudi Arabia; ralshaman@ut.edu.sa (R.A.); wkhubrni@ut.edu.sa (W.S.K.); 2 Department of Pharmacology and Toxicology, College of Pharmacy, Taif University, P.O. Box 21944, Taif 21944, Saudi Arabia; ys.althobaiti@tu.edu.sa; 3 Addiction and Neuroscience Research Unit, Taif University, Taif 21944, Saudi Arabia; 4 Department of Pharmaceutics, Faculty of Pharmacy, University of Tabuk, Tabuk 47713, Saudi Arabia; gh.soliman@ut.edu.sa; 5 Department of Pharmacology, Faculty of Medicine, Assuit University, Assuit 71515, Egypt; hsalama@ut.edu.sa; 6 Department of Pharmacology, Faculty of Medicine, University of Tabuk, Tabuk 47713, Saudi Arabia; 7 Department of Anatomy and Histology, College of Veterinary Medicine, Gyeongsang National University, Jinju 52828, Republic of Korea; pokoh@gnu.ac.kr; 8 Department of Pharmacology and Toxicology, College of Pharmacy, Prince Sttam Bin Abdul Aziz University, Al-Kharj 11942, Saudi Arabia; n_rehman5@hotmail.com

**Keywords:** ischemic stroke, quercetin, mTOR pathway, NLRP3, Nrf2/HO-1

## Abstract

Stroke ranks as the world’s second most prevalent cause of mortality, and it represents a major public health concern with profound economic and social implications. In the present study, we elucidated the neuroprotective role of quercetin on NLRP3-associated pyroptosis, Nrf2-coupled anti-inflammatory, and mTOR-dependent downstream pathways. Male Sprague Dawley rats were subjected to 72 h of transient middle cerebral artery ischemia, followed by the administration of 10 mg/kg of quercetin. Our findings demonstrated that MCAO induced elevated ROS which were coupled to inflammasome-mediated pyroptosis and altered mTOR-related signaling proteins. We performed ELISA, immunohistochemistry, and Western blotting to unveil the underlying role of the Nrf2/HO-1 and PDK/AKT/mTOR pathways in the ischemic cortex and striatum. Our results showed that quercetin post-treatment activated the Nrf2/HO-1 cascade, reversed pyroptosis, and modulated the autophagy-related pathway PDK/AKT/mTOR/P70S6/P6/eIF4E/4EBP1. Further, quercetin enhances the sequestering effect of 14-3-3 and reversed the decrease in interaction between p-Bad and 14-3-3 and p-FKHR and 14-3-3. Our findings showed that quercetin exerts its protective benefits and rescues neuronal damage by several mechanisms, and it might be a viable neuroprotective drug for ischemic stroke therapy.

## 1. Introduction

Stroke is the world’s second leading cause of death, indicating a substantial public health problem [1]. However, the impact of stroke extends beyond mortality, as nearly 50% of survivors experience long-term disability [2,3]. There are an estimated 25.7 million stroke survivors, resulting in about 6 million fatalities and 100 million compromised disabilities [4]. Consequently, stroke represents a major public health concern with profound economic and social implications. Ischemic stroke occurs due to disrupted blood flow, triggering ischemia and hypoglycemia, and subsequently neuronal death [5,6]. Thus, a brief interruption of blood flow for as little as 30 s initiates a harmful cycle called the ischemic cascade, where injured brain cells accumulate ions and release excitatory neurotransmitters [5,6].

Inflammasomes are a critical component of neuroinflammation, and activation thereof is a fundamental trigger for the activation of pyroptosis [7]. An inflammasome, an intricate assembly of proteins, comprises inflammasome sensors known as pattern recognition receptors (PRRs), which can be pathogen-associated molecular patterns (PAMPs) and damage-associated molecular patterns (DAMPs) [8,9]. Typically, an inflammasome comprises NLRs, apoptosis-associated speck-like proteins containing a caspase activation (ASC), and a pro-caspase. There is mounting evidence that the NLRP3 inflammasome contributes to the pathogenesis of neurodegenerative diseases by triggering a chronic inflammatory response that kills off neurons [10]. Furthermore, NLRP3 mediates the cleavage of Gasdermin D (GSDMD), and this process results in the formation of membrane openings which mediate pyroptosis and the subsequent release of mature IL-1 and IL-18 [11]. Thus, treatment strategies that focus on NLRP3-dependent pyroptosis could be promising.

Akt, a protein kinase, acts as an upstream regulator by phosphorylating various crucial regulatory proteins involved in important cellular processes such as ribosomal biogenesis, protein synthesis, and cell proliferation [12]. Some of the key proteins phosphorylated by Akt include the mammalian target of rapamycin (mTOR), p70S6 kinase, and 40S ribosomal protein S6 (S6) [12,13]. The mTOR/p70S6 kinase pathway regulated by Akt plays a vital role in controlling protein synthesis and promoting cell growth. In addition to its role in protein synthesis and cell proliferation, Akt also phosphorylates other proteins that are involved in regulating cell survival and apoptosis. For instance, Akt phosphorylates the forkhead transcription factor (FKHR) and Bad, leading to a reduction in their pro-apoptotic actions [14,15]. Phosphorylation of FKHR and Bad by Akt prevents their pro-apoptotic functions, thereby promoting cell survival. Furthermore, phosphorylated forms of Bad and FKHR interact with a protein called 14-3-3 which has anti-apoptotic properties. This interaction with 14-3-3 further modulates the apoptotic signaling pathways, ultimately promoting cell survival [16].

Quercetin (Qur) is a potent antioxidant, and its antioxidant and anti-inflammatory properties are responsible for its neuroprotective effects in cerebral ischemia. Notably, quercetin’s amphiphilic nature facilitates its rapid penetration through the blood–brain barrier (BBB), enabling a wide range of intracellular protein interactions within the CNS [17,18]. We recently revealed a multimechanistic mechanism by which quercetin affects the expression of numerous proteins in an ischemic brain [19]. In response, Chirumbolo et al. submitted an editorial response that quercetin may induce autophagy in brain ischemia [20]. The study of autophagy behavior in ischemic stroke is a complex issue due to conflicting findings in the published studies. While some studies support the involvement of autophagy, others present opposing evidence [21,22,23]. The objective of this work was to investigate the neuroprotective effects of quercetin in a rat model of middle cerebral artery occlusion and to examine the impact of quercetin on NLRP3-associated pyroptosis, Nrf2-mediated anti-inflammatory response, and mTOR-dependent downstream pathways.

## 2. Results

### 2.1. Quercetin Enhanced Neuronal Cell Viability

**Figure 1:**
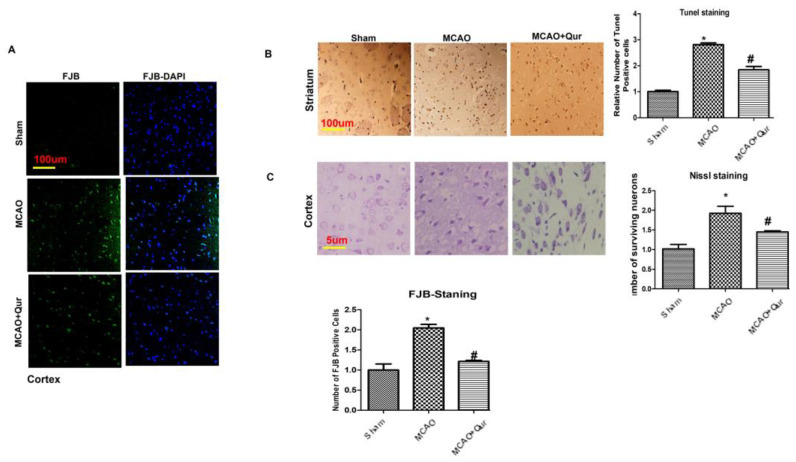
Quercetin attenuated neuronal cell death: (**A**) Images illustrating the immunofluorescence staining of FJB in the cortex (*n* = 5/group) pictured at magnification 10×, scale bar = 100 μm. (**B**) Representative TUNEL histochemistry pictures demonstrate apoptotic cells in the striatum; scale bar = 100 μm. The administration of MCAO resulted in notable neuronal apoptosis, whereas the application of quercetin exhibited a mitigating effect on apoptotic injury. The data reported are related to sham (*n* = 5/group). (**C**) Panels of Nissl staining to evaluate neuronal viability, scale bar = 50 μm. The symbol * is relative to the sham group while the symbol #—to the MCAO group.

To examine the potential neuroprotective effects of quercetin, TUNEL staining and FJB staining were performed. In MCAO animals, both the cortex and the striatum exhibited a high number of FJB-positive cells (Figure 1A, * *p* < 0.05) and TUNEL-positive cells (Figure 1B, * *p* < 0.05), respectively, which were reduced by quercetin (# *p* < 0.05). Furthermore, the administration of quercetin led to a significant increase in the number of viable neurons in the cortex (Figure 1C, # *p* < 0.05 relative to MCAO, * *p* < 0.05) as shown by Nissl staining. 

### 2.2. Effect of Quercetin on ROS

**Figure 2:**
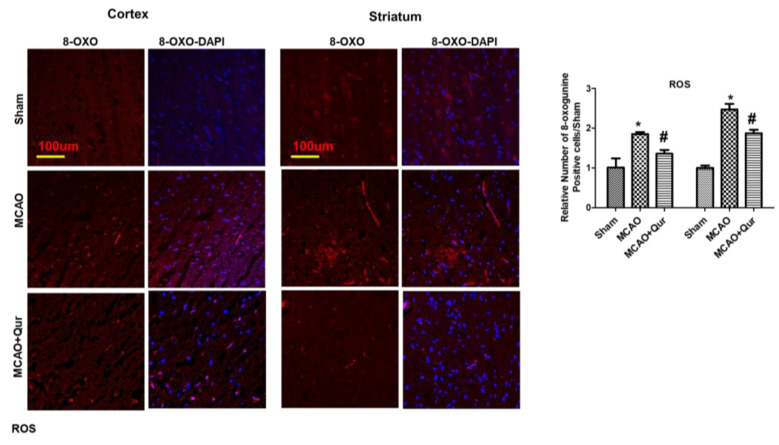
Quercetin attenuated 8-oxo guanine in the MCAO model. 8-oxo guanine was used as a ROS marker. Representative 8-oxo guanine histochemistry pictures demonstrate ROS in the cortex and the striatum (*n* = 5) pictured at magnification 10×, scale bar = 100 μm. Rhodamine was employed for detecting 8-oxo guanine, whereas blue coloration (DAPI) was indicative of nuclear staining. The statistical analysis involved the presentation of data as the means ± SEM. The symbol * is relative to the sham group while the symbol #—to the MCAO group.

Consistent studies have demonstrated 8-oxo guanine as an oxidative stress marker [24]. To further validate this, we performed an immunofluorescence analysis for 8-oxo guanine (Figure 2). Our findings showed that the 8-oxo guanine expression was increased in MCAO (Figure 2, * *p* < 0.05) while quercetin reduced this rise in expression (# *p* < 0.05, Figure 2) both in the cortex and the striatum.

### 2.3. Quercetin-Activated PDK/AKT/mTOR/P70S6/P6/eIF4E/4EBP1

**Figure 3:**
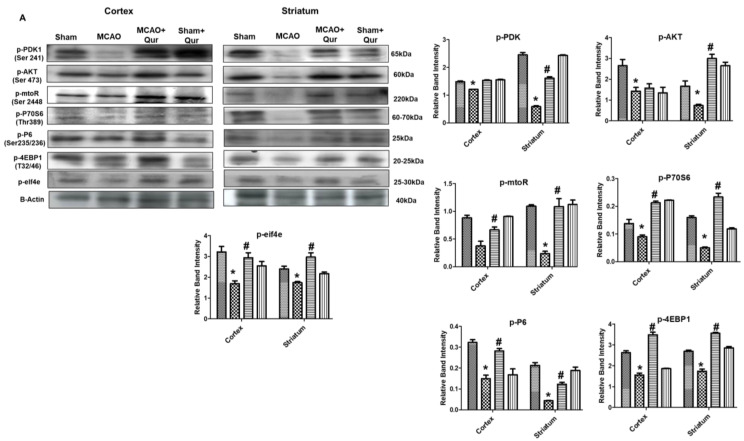

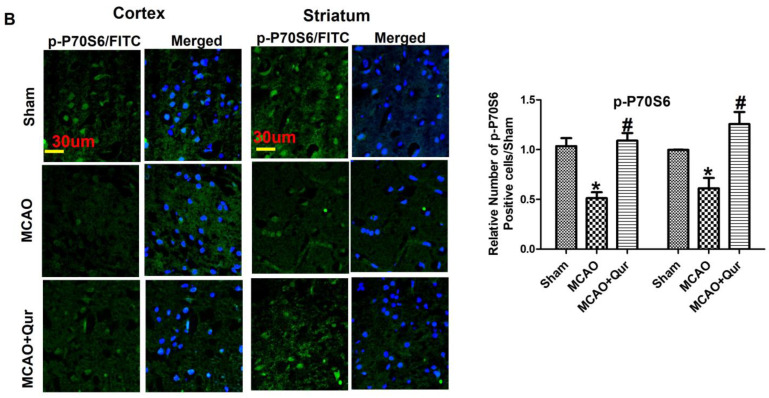
Quercetin modulates the mTOR pathway. (**A**) Western blot analysis of p-PDK, p-AKT, p-mTOR, p-P70S6, p-P6, p-eIF4E, and p-4EBP1 in the cortex and the striatum. The Western blot bands were quantified by ImageJ and the densitometric analysis was quantified relative to β-actin. (**B**) Representative p-P70S6 histochemistry pictures in the cortex and the striatum (*n* = 5) pictured at magnification 40×, scale bar = 30 μm. FITC was employed for detecting p-P70S6, which showed cytoplasmic localization, whereas blue coloration (DAPI) was indicative of nuclear staining. The statistical analysis involved the presentation of data as the means ± SEM. The symbol * is relative to the sham group while the symbol #—to the MCAO group.

Excessive autophagy could exacerbate brain ischemia, and several research articles indicate that mTOR signaling exerts neuroprotective effects by inhibiting autophagy. To investigate this, mTOR and P70S6 protein levels were assessed by Western blotting. The phosphorylated form of these proteins was decreased in the MCAO group (Figure 3A, * *p* < 0.05) as compared to the sham. Moreover, quercetin was found to mitigate the MCAO effect on these protein levels (Figure 3, # *p* < 0.05). To further validate this, we performed an immunofluorescence analysis of p-P70S6, and similar results were demonstrated (Figure 3B, * *p* < 0.05) in quercetin-treated groups (Figure 3B, # *p* < 0.05). Phosphorylation of various downstream targets, including 40S ribosomal protein S6 (S6), is tightly regulated by the p70S6 kinase. This, in turn, regulates the activity of eukaryotic initiation factor 4E (eIF4E)-binding protein 1 (4EBP1), leading to subsequent effects on protein synthesis and cellular growth. The p-S6, p-eIF4E, p-4EBP1 levels were decreased in the MCAO group (Figure 3A, * *p* < 0.05) as compared to the sham. Moreover, quercetin was found to mitigate the p-S6, p-eIF4E, and p-4EBP1 protein levels.

### 2.4. Quercetin Enhances the 14-3-3 Sequestering Effect

**Figure 4:**
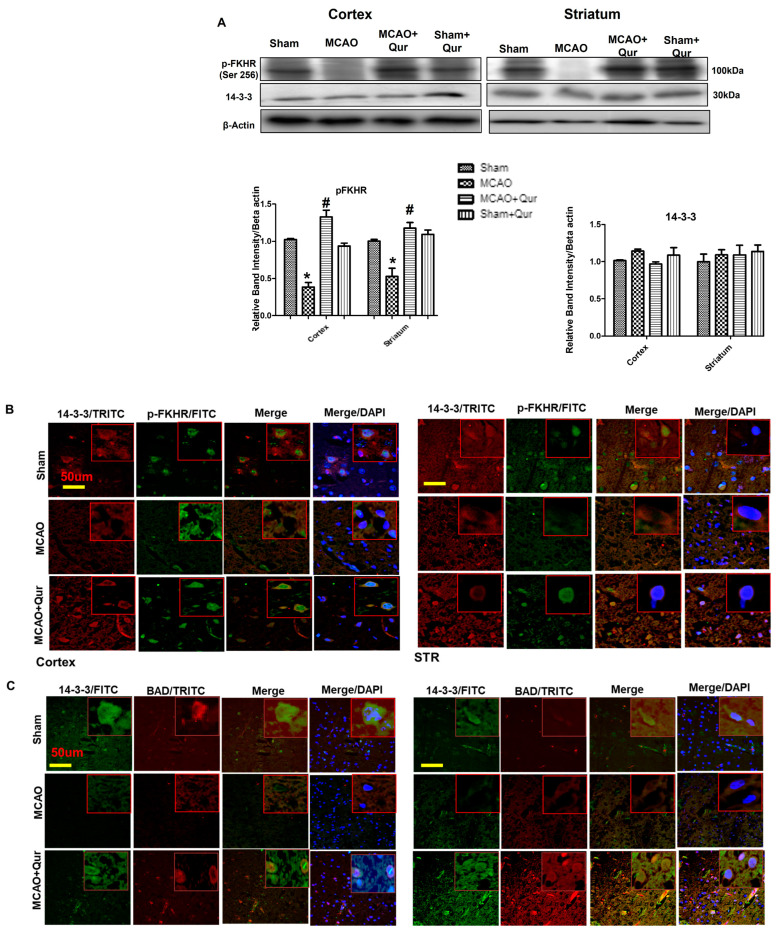
Quercetin dimerizes p-Bad and p-FKHR with 14-3-3. (**A**) Western blot analysis of p-FKHR and 14-3-3 in the cortex and the striatum. The Western blot bands were quantified by ImageJ and the densitometric analysis was quantified relative to beta-actin. The symbol * is relative to the sham group while the symbol #—to the MCAO group. (**B**) Representative p-FKHR and 14-3-3 co-localization histochemistry pictures in the cortex and the striatum (*n* = 5) pictured at magnification 40×, scale bar = 50 μm. FITC and TRITC were employed for detecting p-FKHR and 14-3-3, respectively, and both showed cytoplasmic localization, whereas blue coloration (DAPI) was indicative of nuclear staining. (**C**) Representative p-BAD and 14-3-3 co-localization histochemistry pictures in the cortex and the striatum (*n* = 5), pictured at magnification 40×, scale bar = 50 μm. FITC and TRITC were employed for detecting 14-3-3 and p-BAD, respectively, and both showed cytoplasmic localization, whereas blue coloration (DAPI) was indicative of nuclear staining.

The phosphorylation of Bad and forkhead transcription factors (FKHR) by AKT effectively inhibits the apoptotic function of Bad and FKHR. This occurs through the formation of p-Bad and p-FKHR dimers with 14-3-3, preventing their interaction with Bcl-x(L) and Fas ligand, respectively, and preventing apoptosis [25,26]. To demonstrate the effects of quercetin, we conducted a Western blot analysis in the cortex and the striatum. Our findings showed that the levels of p-FKHR were significantly reduced in the MCAO group (Figure 4A, *p* < 0.01, # *p* < 0.05) compared to the sham group, while no significant effect on the 14-3-3 level was observed. However, quercetin treatment was observed to mitigate the reduction of p-FKHR protein levels (Figure 4A). To further examine the sequestering effects of 14-3-3 on p-Bad and p-FKHR, we performed a double immunofluorescence analysis, examining the co-localization of 14-3-3 with BAD (Figure 4C) and p-FKHR (Figure 4B). These results suggest that quercetin enhances the sequestering effect of 14-3-3.

### 2.5. Quercetin Augmenting Nrf2 Expression Coupled to the Downregulation of Neuroinflammation

**Figure 5:**
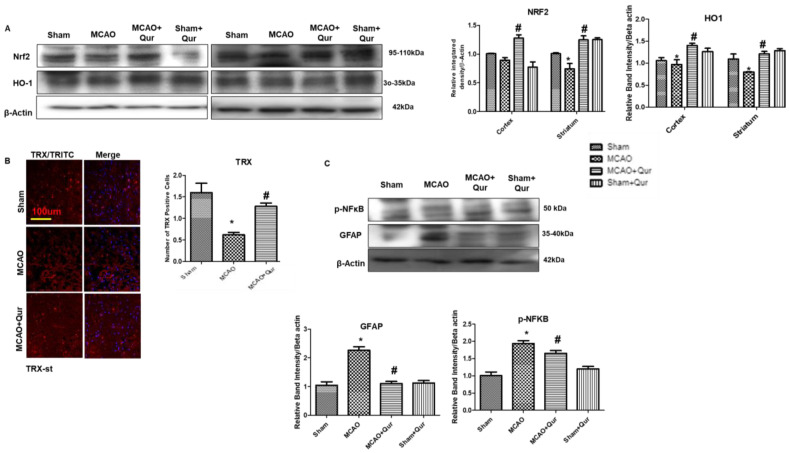
Quercetin augments the Nrf2/HO-1 expression. (**A**) Western blot analysis of Nrf2 and HO-1 in the cortex and the striatum. The Western blot bands were quantified by ImageJ and the densitometric analysis was quantified relative to beta-actin. (**B**) Representative TRX histochemistry pictures in the striatum (*n* = 5) pictured at magnification 10×, scale bar = 100 μm. TRITC was employed for detecting TRX and showing cytoplasmic localization, whereas blue coloration (DAPI) was indicative of nuclear staining. (**C**) Western blot analysis of p-NF-κb and GFAP in the cortex. The Western blot bands were quantified by ImageJ and the densitometric analysis was quantified relative to beta-actin. The statistical analysis involved the presentation of data as the means ± SEM and the utilization of two-way ANOVA with post-hoc Tukey’s test; * *p* < 0.05 is compared to the sham while # *p* < 0.05 is compared to MCAO.

To assess the quercetin’s antioxidative properties, we evaluated Nrf2, HO-1, and thioredoxin (TRX) levels (Figure 5A). The Western blot revealed that there were alterations in Nrf2 and HO-1 in the MCAO group (Figure 5A). The administration of quercetin led to a significant elevation in the levels of Nrf2 and HO-1 (Figure 5A, # *p* < 0.05). To further support our findings, we performed immunofluorescence analysis of thioredoxin (TRX), a small-molecular-weight antioxidant protein, and similar results were demonstrated (Figure 5B, * *p* < 0.05, # *p* < 0.05). These results suggest that quercetin mitigated an MCAO-induced oxidative stress, at least in part, by activating Nrf2, which in turn regulates the production of downstream antioxidants. Additionally, activation of the NF-κB pathway plays a crucial role in inflammatory cascades. To investigate this further, a Western blot analysis was conducted to measure the protein levels of p-NF-κB and GFAP in the brain cortex (Figure 5C). The results demonstrated an increase in the expression of these proteins following MCAO (* *p* < 0.05).

### 2.6. Quercetin Attenuated MCAO-Induced NLRP3-Dependent Pyroptosis

**Figure 6:**
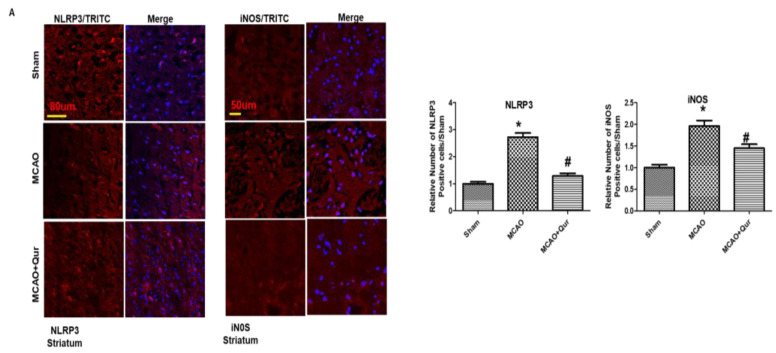

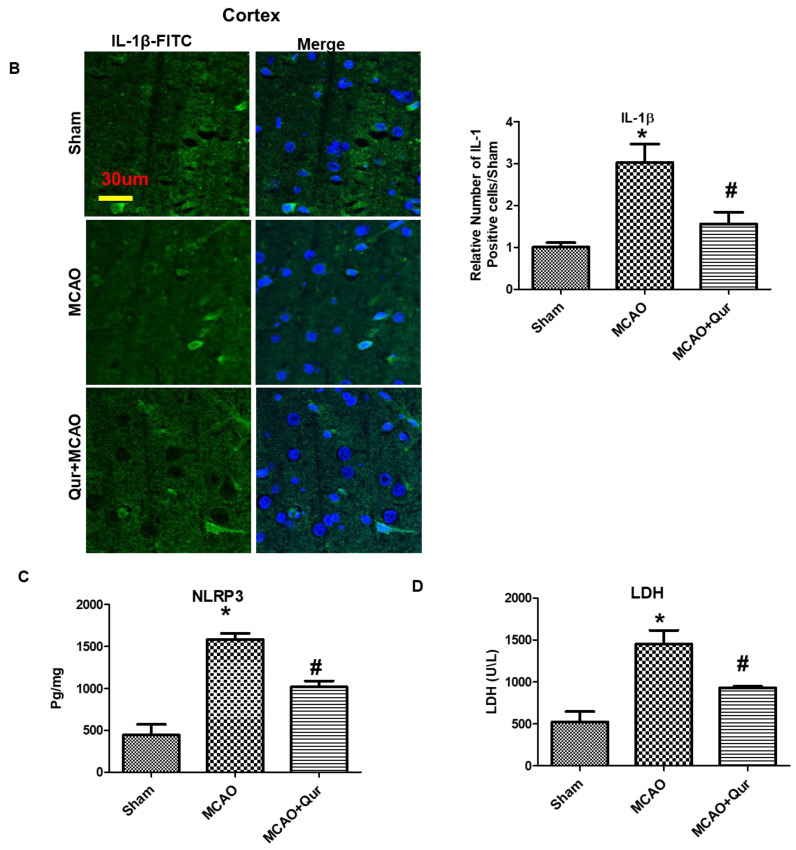
Quercetin administration reversed pyroptosis by the NLRP3 pathway. (**A**) Pictures of NLRP3 (scale bar = 80 um) and iNOS (scale bar = 50 um) in the striatum. MCAO induced the expression of these markers while quercetin treatment attenuated the expression level. Both NLRP3 and iNOS represent cytoplasmic localization. TRITC was employed for detecting iNOS and NLRP3, whereas blue coloration (DAPI) was indicative of nuclear staining. The statistical analysis involved the presentation of data as the means ± SEM and the utilization of one-way ANOVA with post-hoc Tukey’s test; * *p* < 0.05 is compared to the sham while # *p* < 0.05 is compared to MCAO. (**B**) IL-1β expression in the cortex, magnification 40×, scale bar = 30 μm. FITC was employed for detecting IL-1, whereas blue coloration (DAPI) was indicative of nuclear staining. The statistical analysis involved the presentation of data as the means ± SEM and the utilization of two-way ANOVA with post-hoc Tukey’s test; * *p* < 0.05 is compared to the sham while # *p* < 0.05 is compared to MCAO. (**C**) ELISA analysis of NLRP3 in the cortex. The symbol * is relative to the sham group while the symbol #—to the MCAO group. (**D**) Serum LDH evaluation for demonstrating leakage in the cell membrane. The symbol * is relative to the sham group while the symbol #—to the MCAO group.

To demonstrate the anti-pyroptosis effect of quercetin in MCAO, we determined the levels of NLRP3, IL-1β, and iNOS (Figure 6A,B) by immunostaining. We demonstrated here that NLRP3, IL-1β, and iNOS were upregulated in MCAO (* *p* < 0.05) while downregulated by quercetin (# *p* < 0.05). Further, we performed ELISA to determine the expression of NLRP3 in the cortex (Figure 6C). Compared with the sham group, the level of NLRP3 was upregulated in the MCAO group (* *p* < 0.05), which was reversed by quercetin treatment (# *p* < 0.05, Figure 6C). Moreover, the elevated LDH level demonstrated the compromised integrity of the cell membrane leading to pyroptosis and the release of mature IL-1β (Figure 6D). 

## 3. Discussion

In the current study, we showed that post-treatment with quercetin alleviated MCAO-induced ischemic damage by activating the Nrf2 pathway and downregulating inflammasome-coupled pyroptosis, which were likely contributing mechanisms to this neuroprotection. Further, the upregulation of Nrf2 was associated with a reduction in the amount of 8-oxo guanine and FJB-positive cells in the cortex and the striatum. Moreover, quercetin modulated the PDK/Akt/mTOR/P70S6/P6/eIF4E/4EBP1 pathway. Previous studies demonstrated that autophagy inhibits mTOR suppression [27], which may suggest that in our study quercetin prevented MCAO-induced neurodegeneration at least in part by inhibiting autophagy and promoting PI3K/AKT/mTOR.

There is growing interest in the use of Nrf2 activators for clinical applications since the Nrf2/ARE signaling pathway has emerged as a possible therapeutic target for reducing oxidative stress [28,29]. Using Western blotting, we looked at the protein levels of Nrf2 and HO-1 to determine the possible antioxidative effect of quercetin. Our results demonstrated that quercetin inhibited ROS while enhancing Nrf2 and HO-1 protein expression. In our previous study using a rat model of ischemic stroke, we observed that inhibiting Nrf2 with ATRA increased the infarction area [30]. Additionally, according to Martin-de-Saavedra et al., Nrf2 modulated the levels of neurotransmitters such as dopamine, noradrenaline, and serotonin, while Nrf2-mutant animals demonstrated elevated glutamate levels [31]. Moreover, the deviant behavioral outcomes observed in the mice lacking the Nrf2 gene offer additional evidence supporting the involvement of Nrf2 in neurodegenerative disorders [32]. Furthermore, the excessive generation of free radicals plays a critical role in the activation of NF-κB transcription factors [33]. Our results demonstrated that an increase in ROS levels coincided with elevated NF-κB levels, indicating the presence of a free radical-mediated cellular damage. Moreover, it is well-established that the NLRP3 inflammasome plays a significant role in the progression of neurodegeneration during ischemic stroke. Thus, targeting the NLRP3 inflammasome emerges as a plausible strategy for the development of anti-ischemic stroke medications [34,35]. In this study, we observed a significant rise in the expression of pyroptosis markers. Similar to other forms of cellular death, pyroptosis is initiated in response to oxidative stress triggered by disruptions to the cellular redox equilibrium [36]. The ROS content was shown to significantly increase in our study, and this increase was positively connected with increased pyroptosis, suggesting that oxidative stress is a major contributing factor driving pyroptosis in ischemic stroke. Moreover, the upregulation of IL-1β, the presence of FJB-positive cells, and the elevated LDH level were reversed by quercetin.

Previously published research suggested that the PI3K/AKT pathway is important in reducing ischemic damage [37]. Additionally, mTOR stimulation by AKT is essential for autophagy suppression [38] and genetic or pharmacologic suppression of mTOR signaling can trigger autophagy [39]. Furthermore, inhibition of the PI3K/AKT/mTOR pathway has been linked to the activation of autophagy and apoptotic cell death [40]. Uchiyama’s research group reported that in a mouse model of ischemia–hypoxia, numerous damaged neurons exhibited characteristics of autophagic/lysosomal cell death [41]. In previous studies, several natural drugs were shown to exhibit neuroprotective effects by downregulating autophagy in ischemic stroke [42,43]. While our study did not yield direct evidence of quercetin’s impact on autophagy, the observation of reduced mTOR phosphorylation during autophagy activation suggested that quercetin may play a role in modulating excessive autophagy in our experimental model. The mTOR/p70S6 kinase pathway plays a crucial role in the regulation of protein synthesis and the modulation of cellular growth [44]. Previous research indicated that the levels of phosphorylated mTOR and p70S6 kinase exhibit a decline in ischemic brain injury and Alzheimer’s disease [45]. Furthermore, the Akt/mTOR/p70S6 kinase signaling pathway is known to have a significant impact on safeguarding the heart from infarction [46]. In addition, it has been observed that the inhibition of translation initiation occurs through dephosphorylation of eIF2B as a result of dephosphorylation of mTOR and p70S6 kinase [47,48,49].

Protein 14-3-3 plays a role in inhibiting apoptosis by binding to pro-apoptotic molecules such as Bad and FKHR [25,26]. Prior research indicated that the application of apoptotic stimuli leads to dephosphorylation of Bad, which consequently causes its separation from 14-3-3. This process ultimately triggers the activation of Bax and caspase-3. Considering the crucial role of the interaction between p-Bad and 14-3-3 in preventing cellular apoptosis, we hypothesize that alterations in protein–protein interactions may contribute to the neuroprotective effects of quercetin. We demonstrated here that quercetin reversed the reduction of the interaction between p-Bad and 14-3-3 and between p-FKHR and 14-3-3.

There are also limitations in our study. Extensive molecular approaches will unveil the underlying mechanisms. Further, a key marker of autophagy, i.e., the ratio of microtubule-associated protein 1 light chain 3B (LC3B) II/I, was missed in this study. Moreover, some studies suggested the cross-talk of pyroptosis and autophagy, while some suggested their opposite role.

## 4. Materials and Methods

### 4.1. Animal Grouping and Drug Treatment

The study utilized male rats with a weight range of 190–230 g and 8–9 weeks of age which were procured from the National Institute of Health (NIH) located in Islamabad, Pakistan. Prior to the initiation of the experiment, the rats underwent an acclimation period. The study obtained ethical approval from the REC-RIPS internal review board under reference number REC/RIPS/Cology/16-2019. The groups were categorized as follows: sham-operated control group, MCAO control group, MCAO + Qur group, and sham + Qur group. Quercetin (Sigma Aldrich; USA Catalogue No: Q4951) was dissolved in phosphate-buffered saline containing 0.05% dimethyl sulfoxide as a solvent. It was administered at a dose of 10 mg/kg 90 min after ischemia and subsequently for two consecutive days intraperitoneally [19]. Following absorption, quercetin undergoes metabolism in various organs, and the resulting metabolites can be detected in plasma in the form of glucuronide or sulfate [50]. Additionally, both quercetin and its metabolites can traverse the blood–brain barrier [51]. The primary metabolite, quercetin glucuronide, is transported through plasma to target tissues where it exerts its biological effects [52].

### 4.2. tMCAO Surgery

The surgery was performed as per our previous experiments [53]. The rodents were anesthetized with a combination of xylazine and ketamine administered intraperitoneally (I/P) turned the rodents anesthetize. Careful dissection of the surrounding tissues allowed for the identification and release of the right common carotid artery (CCA). Alongside the CCA, the vagus nerve was cautiously released. To ensure proper hemostasis, the occipital and superior thyroid arteries, which are minor branches of the external carotid artery, were ligated using black silk (6/0) and coagulated. A permanent knot was secured above the origin of the superior thyroid artery at the hyoid bone. Next, the external carotid artery was cut near its bifurcation using sharp scissors. To induce occlusion of the middle cerebral artery (MCA), a 3 cm long nylon silk thread (3/0) was inserted from the external carotid artery and advanced towards the internal carotid artery. The advancement of the thread was carefully monitored, and a slight resistance indicated successful occlusion of the MCA. 90 min after ischemia the nylon suture was removed, and the channel was recanalized. All the animals were euthanized 72 h following reperfusion to obtain samples. The sham group was subjected to comparable procedures but without the use of nylon. During the study, seven rats died, including four from the MCAO group and three from the MCAO + Qur group, which were eliminated from the research. This method is commonly employed in experiments, although a significant drawback of this model is the risk of subarachnoid hemorrhage owing to vascular rupture and overheating. At the end of the experimental procedure, the animals were euthanized by placing them in a CO_2_ chamber. Brain tissue was collected and either preserved in 4% paraformaldehyde at 4 °C for morphological analysis and/or dissected carefully to isolate the ipsilateral cortex and the striatum and kept at 70 °C for biochemical analysis.

### 4.3. TUNEL Staining

TUNEL staining was performed according to the manufacturer’s instructions using the DNA Fragmentation Detection Kit (Oncogene Research Products, Cambridge, MA, USA). After performing the procedure, JPG images were captured using a light microscope, and the TUNEL-positive cells were quantified using the ImageJ program (NIH, USA) by adjusting the TIF images to the same threshold intensity for all groups. The results were presented as the relative number of TUNEL-positive neuronal cells per section compared to the control samples.

### 4.4. Fluoro-Jade B (FJB) Staining

After the deparaffinization process, the slides underwent immersion in a 1% NaOH solution, followed by a sequence of ethanolic solutions with varying degrees of dilution. Following this, the slides underwent a rinsing process and were treated with KMNO4, acetic acid, and FJB. The quantification was executed utilizing the ImageJ software version 1.30, as previously discussed [54].

### 4.5. Western Blotting

The specimens were lysed in a lysis buffer, and quantification of the protein content in the homogenate was performed using a BioRad protein assay kit. In the experimental procedure, 30 μg of the protein were applied to 10% SDS-PAGE gels and subjected to electrophoresis, and after protein separation, PVDF membranes (Millipore, Billerica, MA, USA) were utilized for protein transfer via immunoblotting. The antibodies utilized in this study were anti-PDK1 Ser 241 (3061), anti-p-P70S6 Thr389 (9205), anti-p-P6 Ser235/236 (3061), anti-p-4EBP1T32/46 (2855), anti-p-elf4e (9741) procured from Cell Signaling and anti-14-3-3 (Sc-1657), anti-p-FKHR Ser 256 (SC-101681), anti-p-NFκB (SC-271908), anti-Nrf2 (SC-722), anti-HO1 (SC-136961), anti-p-AKT Ser 473 (SC-SC-798), anti-p-mTOR Ser 2448 (SC-293132), and anti-β-Actin from Santa Cruz Biotechnology, California, USA, all employed at a dilution of 1:1000. Subsequently, the membranes were subjected to incubation with secondary antibodies and the protein bands were observed through the employment of an ECL detection reagent. The X-ray films were subjected to scanning, and the optical densities of the bands were quantified relative to the corresponding β-actin band through the utilization of the ImageJ software, as previously explicated [55].

### 4.6. Immunofluorescence

For immunofluorescence, the same protocols were used except fluorescent secondary antibodies were used, the slides were mounted with a fluorescent mounting medium, and no ABC reagent and terminal dehydration were used. The antibodies used were p-P70S6 (SC-8416), 14-3-3 (Sc-1657), p-FKHR Ser 256 (SC-101681), GFAP (SC-33673) purchased from Santa Cruz, while NLRP3 (A5652) was from ABclonal, China. Anti-8-oxoguanine was a kind gift (and were purchased from Millipore, USA) [56].

### 4.7. Enzyme-Linked Immunosorbent Assay for NLRP3

The quantification of NLRP3 was performed following the manufacturer’s guidelines. Tissue specimens weighing approximately 50–70 mg were homogenized, and the resulting supernatant was collected. The concentration of NLRP3 (CAS#A5652, Shanghai MLBIO Biotechnology Co., Ltd., Shanghai, China) was determined using an ELISA microplate reader and employing methods discussed in previous studies [30].

### 4.8. Serum Lactate Dehydrogenase (LDH) Analysis

The determination of serum lactate dehydrogenase (LDH) was performed using the Teas principle. In this method, LDH facilitates the conversion of pyruvate to lactate while simultaneously oxidizing NADH to NAD. The rate of NADH oxidation can be quantified by measuring the decrease in absorbance at 340 nm. This rate is directly correlated to the LDH activity present in the serum.

### 4.9. Statistical Analysis

The quantification of Western blot bands and morphological data was performed using the ImageJ software (version 1.30; available for download at https://imagej.nih.gov//, accessed on 10 July 2023) and analyzed with the GraphPad Prism 5 software. The data were presented as the means with the standard error of the mean (SEM). Statistical analysis was conducted using one-way and two-way ANOVA, followed by a post-hoc Tukey’s multiple comparison test, using the GraphPad Prism 5 software. Statistically significant differences were indicated by symbols * or #, with a significance level of *p* < 0.05.

## 5. Conclusions

We demonstrated that post-treatment with quercetin reduced the oxidative stress-associated inflammatory processes and activated Nrf2/HO-1 and mTOR-related signaling. Our findings suggest that quercetin attenuated MCAO-induced neuronal damage at least in part by PDK/AKT/mTOR/P70S6/P6/eIF4E/4EBP1 signaling. Further studies are required to elucidate the underlying mechanism of quercetin in cerebral ischemia.

## Data Availability

The data will be available from the corresponding author upon request.
